# Supplementary material for: Prefrontal projections to the thalamic nucleus reuniens mediate fear extinction
Source: Nat Commun. 2018 Oct 30;9:4527. doi: 10.1038/s41467-018-06970-z (PMC6207683; doi:10.1038/s41467-018-06970-z)
Supplement: Supplementary file 1 — Supplementary Information [file 41467_2018_6970_MOESM1_ESM.pdf]

Supplemental Figures. Ramanathan, K.R., Jin, J., Giustino, T. F., Payne, M. R., and Maren, S. Prefrontal projections to the thalamic nucleus reuniens mediate fear extinction. *Nature Communications.*

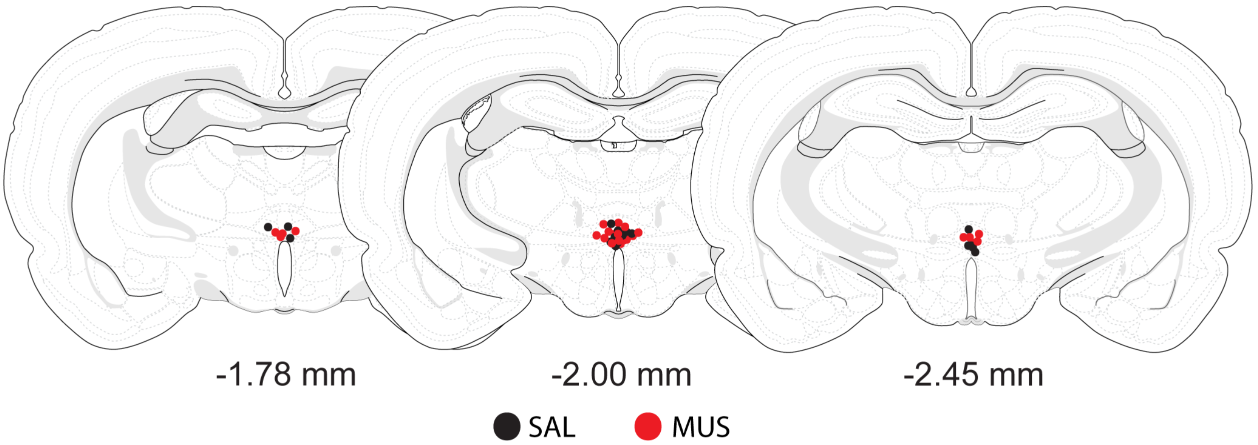

Supplemental Figure 1. Representative brain atlas images showing the cannula tip of the animals included in the analysis at three different levels in the anterior-posterior axis. The distribution of cannula placements was similar across all groups and all experiments. Illustrations are original artwork adapted from open access brain atlas templates[1].

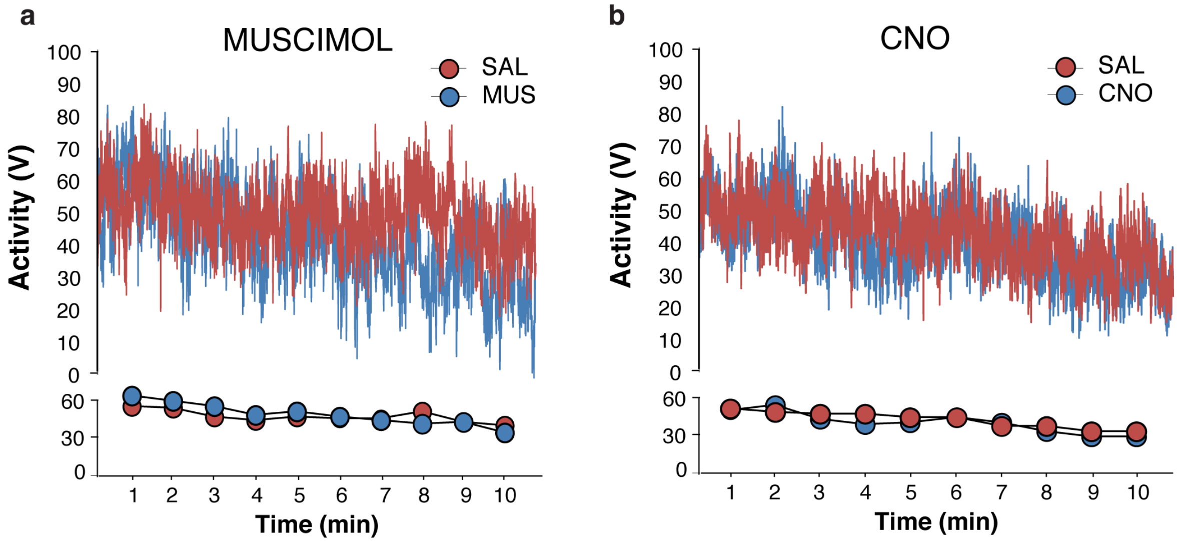

Supplemental Figure 2. Baseline motor activity (top row) and freezing (bottom row) during a 10-minute baseline prior to the extinction retrieval tests upon infusions of saline (red lines and circles) or muscimol (blue lines or circles) in RE (**a**) or systemic administration of saline (red lines and circles) or CNO (blue lines or circles) (**b**). The data represent average load-cell voltages sampled at 5Hz of chamber displacement during the test for all the animals included in the analysis each group; 1-min averages (means ± s.e.m.s.; note that error bars are smaller than the diameter of the plot symbols) of these values are shown below each plot. The activity data show that there are no differences in drug-treated animals in their overall motor activity across the 10-min test, revealing that nonspecific decreases in activity do not account for the increased freezing observed to presentation of the CS after the baseline (the overall decrease in activity across the test occur as animal's habituate their exploratory activity of the context).

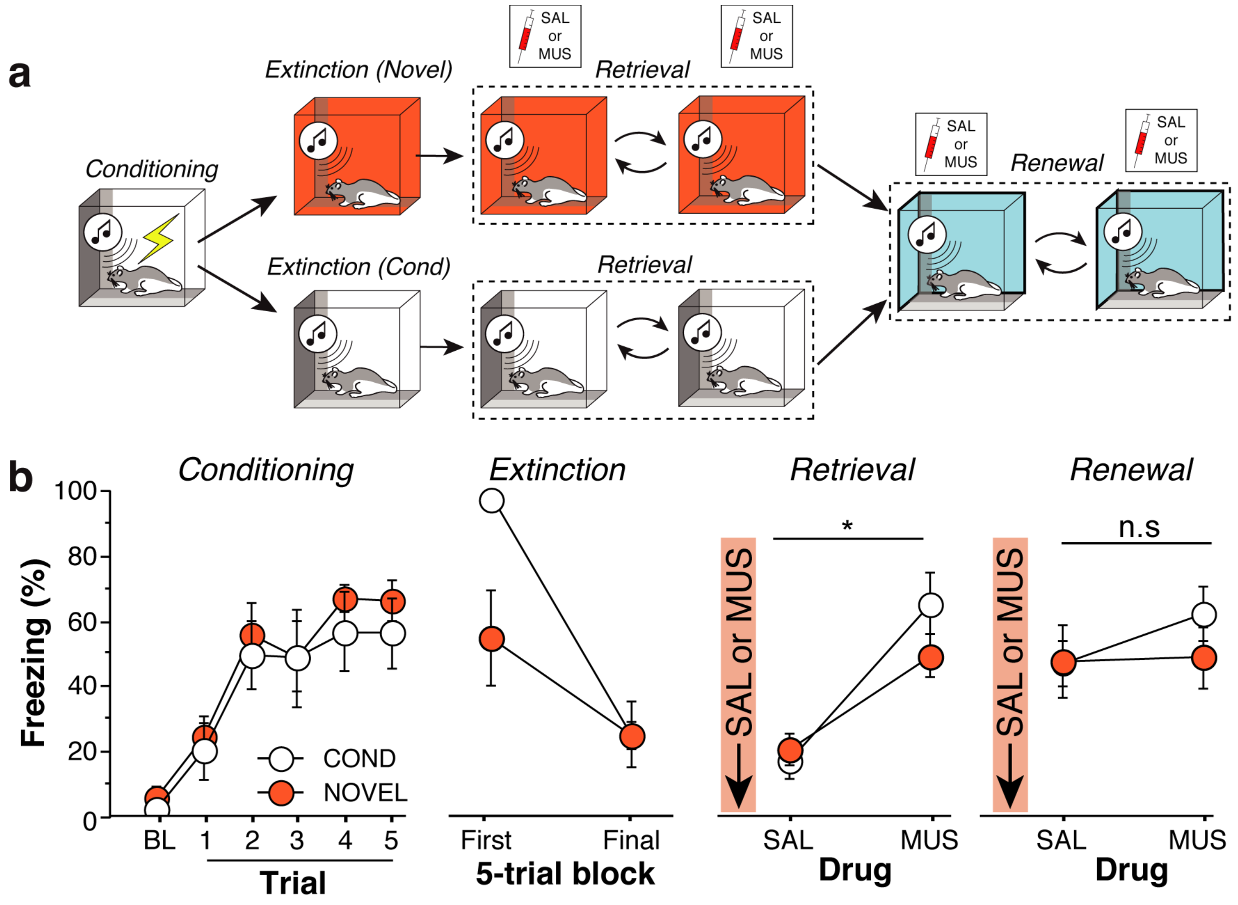

Supplemental Figure 3. RE inactivation impairs extinction retrieval in animals extinguished inside or outside of the conditioning context. **a** Schematized behavioral design. Illustrations are original artwork composed by the authors and adapted from ref[2]. **b** (Conditioning, left), Percentage of freezing during the 3-min baseline (BL) and 1-min interstimulus interval (ISI) following the last CS-US pairing during the fear conditioning session. (Extinction, right), Percentage of freezing during the first and last extinction blocks (each block represents average freezing of 5 ISIs) for the extinction training session. (retrieval), Average percentage freezing during 5 CS test trials during extinction retention tests after either SAL or MUS infusions in RE. (Renewal), Average percentage freezing during 5 CS test trials during renewal tests after either SAL or MUS infusions in RE (COND: n = 6; NOVEL: n = 7). All data are means ± s.e.m.s.

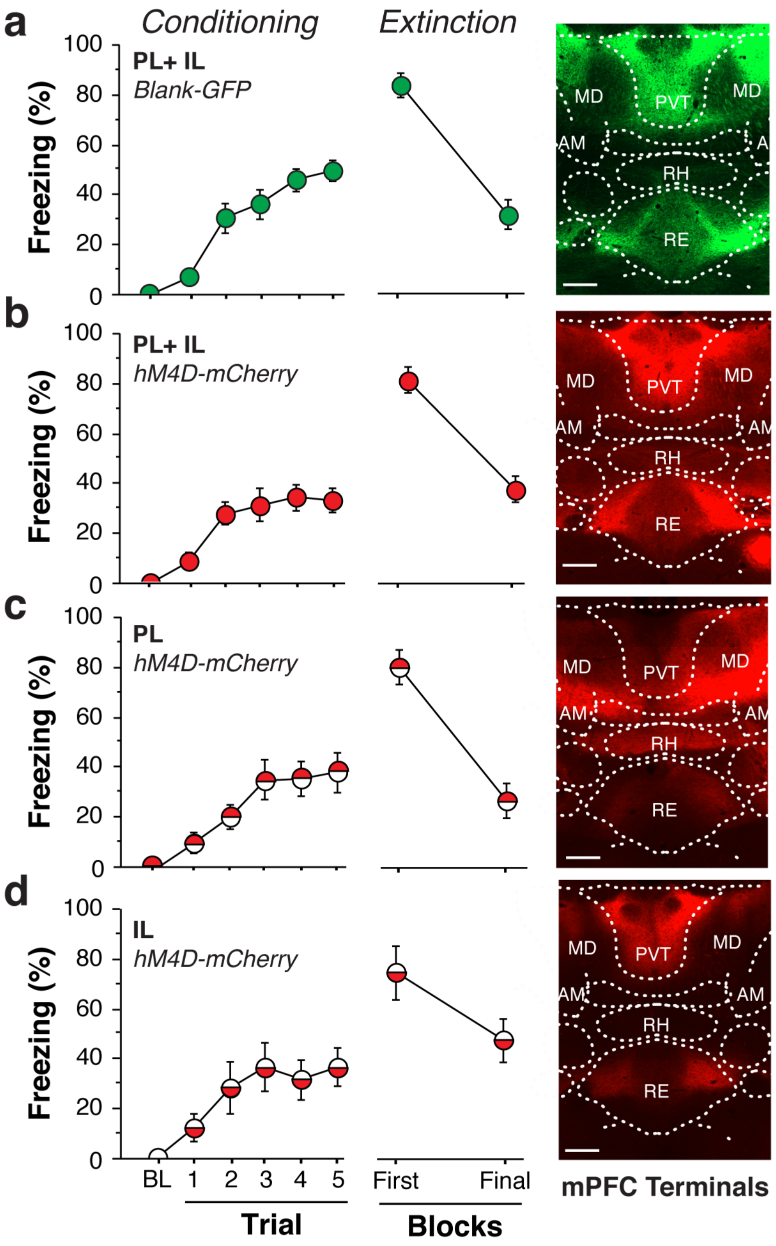

Supplemental Figure 4. Behavioral data and terminal expression in RE after DREADD expression in mPFC. **a-d** (Conditioning, left). Percentage of freezing during the 3-min baseline (BL) and 1-min interstimulus interval (ISI) following the last CS-US pairing during the fear conditioning session. (Extinction, middle), Percentage of freezing during the first and last extinction blocks (each block represents average freezing of 5 ISIs) for the extinction training session for the 4 groups with viral injections in PL or IL or both. (Terminals, right), Representative terminal expression in mid-line thalamus after viral infusions in PL or Il or both. All data are means ± s.e.m.s.

References:

1. Swanson, L. R. Brain maps 4.0—Structure of the rat brain: An open access atlas with global nervous system nomenclature ontology and flatmaps. *J Comp Neurol*, https://doi.org/10.1002/cne.24381 (2017).

2. Marek, R. *et al.* Hippocampus-driven feed-forward inhibition of the prefrontal cortex mediates relapse of extinguished fear. *Nat Neurosci* **21,** 384–392 (2018).
